# Highway Deformation Monitoring by Multiple InSAR Technology

**DOI:** 10.3390/s24102988

**Published:** 2024-05-08

**Authors:** Dan Zhao, Haonan Yao, Xingyu Gu

**Affiliations:** College of Transportation, Southeast University, Nanjing 211100, China; zdan1202@163.com (D.Z.); yao_hn@163.com (H.Y.)

**Keywords:** road deformation, Jiangsu region, InSAR technique, correlation analysis, difference analysis

## Abstract

Addressing the challenge of large-scale uneven deformation and the complexities of monitoring road conditions, this study focuses on a segment of the G15 Coastal Highway in Jiangsu Province. It employs PS-InSAR, SBAS-InSAR, and DS-InSAR techniques to comprehensively observe deformation. Analysis of 73 image datasets spanning 2018 to 2021 enables separate derivation of deformation data using distinct InSAR methodologies. Results are then interpreted alongside geological and geomorphological features. Findings indicate widespread deformation along the G15 Coastal Highway, notably significant settlement near Guanyun North Hub and uplift near Guhe Bridge. Maximum deformation rates exceeding 10 mm/year are observed in adjacent areas by all three techniques. To assess data consistency across techniques, identical observation points are identified, and correlation and difference analyses are conducted using statistical software. Results reveal a high correlation between the monitoring outcomes of the three techniques, with an average observation difference of less than 2 mm/year. This underscores the feasibility of employing a combination of these InSAR techniques for road deformation monitoring, offering a reliable approach for establishing real-time monitoring systems and serving as a foundation for ongoing road health assessments.

## 1. Introduction

The factors mentioned, including prolonged construction, outdated underground pipe network designs, and underground space development, contribute to significant road deformations, impairing their functionality and safety. Consequently, prioritizing road deformation monitoring and research holds substantial theoretical and practical importance. Such endeavors are instrumental in realizing the vision of a robust transportation infrastructure and advancing towards a modern, comprehensive transportation system.

Traditional methods of monitoring road deformation primarily rely on ground-based observation and measurement, characterized by high costs, lengthy monitoring periods, and limited spatial coverage. However, in recent years, there has been a growing focus on utilizing interferometric synthetic aperture radar (InSAR) technology to study surface and structural deformation. InSAR leverages the phase information from synthetic aperture radar (SAR) to conduct interferometric processing of multi-temporal phases, enabling high-precision measurement of surface deformation. Furthermore, to address technical limitations and accommodate diverse detection requirements, continuous innovations in InSAR technology have emerged, ranging from D-InSAR to PS-InSAR, DS-InSAR, SBAS-InSAR, among others. These advancements have significantly enhanced the accuracy, detection range, and computational efficiency of deformation monitoring, marking a substantial improvement over traditional methods [1,2].

The breadth of research in deformation monitoring utilizing various InSAR techniques is indeed extensive and continues to evolve. Wang Zhidong et al.’s work on early detection of geologic hazards in the Longmenshan-Daduhe region highlights the comprehensive interpretation of deformation using InSAR techniques [3]. Yu Shuyuan et al.’s comparison of SBAS-InSAR and PS-InSAR for monitoring ground subsidence along a subway line in Hefei City provides valuable insights into the effectiveness of different methodologies [4].

Furthermore, Wu Yan and Liu Bing et al.’s utilization of DS-InSAR and MCTSB-InSAR techniques for monitoring subsidence along transportation infrastructure in Zhengzhou and Beijing respectively contributes significantly to transportation monitoring practices [5,6].

Many domestic and international literature have utilized InSAR technology for a wide range of the observation applications, and their results show that InSAR technology has sufficient accuracy in the field of deformation observation [7,8,9,10,11].

Theoretical advancements in algorithms and techniques also play a crucial role. Kampes et al.’s deformation parameter estimation model based on spatio-temporal three-dimensional de-entanglement and integer least squares (ILS) methods [12]. Pablo et al.’s CPT method for handling high-quality targets [13], Costantini’s PSP method for improving parameter solving accuracy [14,15], and Samsonov’s MSBAS technique for obtaining two-dimensional deformation time series demonstrate the diverse approaches to enhancing monitoring capabilities [16].

Moreover, Zhangfeng Ma et al.’s new data processing method addresses computational efficiency concerns, further enriching the field’s methodological toolkit and facilitating broader applications of InSAR technology [17].

Different InSAR techniques have their own characteristics and advantages. PS-InSAR technique is based on fixed scattering for monitoring, which can detect smaller deformations, but due to the limited number of fixed scatterers, it has limited coverage of deformation monitoring in the overall observation area. DS-InSAR technique can carry out deformation monitoring on a large scale, but it is generally suitable for application scenarios of short-term and small-amplitude deformation monitoring. SBAS-InSAR is also suitable for large-scale deformation monitoring, but its resolution is lower than that of DS-InSAR, which makes it difficult to capture small deformations on the road. By combining the measurement results of multiple InSAR techniques to comprehensively interpret the deformation observation area, the accuracy of the deformation area zones can be improved to a certain extent.

The paper highlights the significant role of InSAR technology in road deformation monitoring and outlines the utilization of PS-InSAR, SBAS-InSAR, and DS-InSAR techniques for this purpose. By focusing on the K843+699-K887+991.5 section of the Jiangsu Coastal Highway, the study conducts settlement observation and data analysis, integrating geological conditions and geomorphological features to interpret deformation results comprehensively.

Furthermore, the paper evaluates the accuracy, reliability, and consistency of the deformation results obtained from different InSAR technologies within the context of road deformation monitoring. By aligning the findings with geological conditions and geomorphological features on the ground, the study provides a holistic understanding of the observed deformations.

Through this approach, the research contributes to advancing the understanding and application of PS-InSAR, SBAS-InSAR, and DS-InSAR techniques in highway monitoring, offering insights into their respective strengths and limitations in assessing road deformations. The integration of previous studies on surface deformation further enriches the analysis, providing a comprehensive overview of the research area’s dynamics and facilitating informed decision-making in infrastructure management and maintenance.

## 2. Materials and Methods

### 2.1. PS-InSAR Technology

The fundamental principle of Persistent Scatterer Interferometric Synthetic Aperture Radar (PS-InSAR) technology entails the selection of one SAR image from the existing M + 1 acquisitions as the main reference image. Subsequently, the remaining SAR images are geometrically registered to the spatial reference frame of the main image, functioning as sub-images. This process enables the creation of M groups of interferometric pairs. These pairs undergo differential interferometric processing, wherein the interferometric phase, denoted as φ, is computed. The formulation for the interferometric phase is as follows:

(1) $$\varphi =\varphi_{ref}\;+\varphi_{top}+\varphi_{def}+\varphi_{atm}\;+\varphi_{noi}$$

In the provided equation, φ represents the interferometric phase of the interfering pair, wherein φ_ref_, φ_top_, φ_atm_, and φ_noi_ correspond to the reference ellipsoid phase, the phase attributed to ground undulation, atmospheric delay, and random noise, respectively. The residual φ_def_ denotes the desired surface settlement phase. To ensure precise estimation of the deformation phase, the elimination of extraneous interfering phases outlined in the equation is crucial. In the context of PS-InSAR methodology, the mitigation of interfering phases primarily relies on leveraging Digital Elevation Model (DEM) data in conjunction with high-precision satellite orbit information [18].

### 2.2. SBAS-InSAR Technology

The foundational concept underlying the SBAS-InSAR technique is predicated on the assumption of having N + 1 pairs of SAR images acquired at distinct time intervals, denoted as t_0_, t_1_,…, t_M_. Subsequent to aligning and resampling all the images to a common spatial reference frame, M interferometric pairs are generated. The condition for the number of interferometric pairs, M, is dictated by the following criteria:

(2) $$\frac{N+1}{2}\leq M\leq \frac{N\left(N+1\right)}{2}$$

If we consider the ith interferometric pair composed of two images acquired at time instances t_A_ and t_B_ (where t_A_ < t_B_), the phase difference in the interferogram at coordinates (x, y) must adhere to the following simplified equation within the corresponding coordinate system:

(3) $$\delta \varphi_{i}(x,y)\approx \frac{4\pi }{\lambda }\left[d_{t_{A}}\left(x,y\right)-d_{t_{A}}\left(x,y\right)\right]$$

Here, λ denotes the radar wavelength, while dt(x,y) signifies the deformation of the point relative to t_0_ at times t_A_ and t_B_, respectively. Ultimately, the deformation phase is derived through a least squares solution employing singular value decomposition [19].

### 2.3. DS-InSAR Technology

The DS-InSAR (Differential Synthetic Aperture Radar Interferometry) technique enhances pixel resolution and offers more comprehensive deformation insights by identifying distributed targets, known as DS points. These DS points typically exhibit weaker coherence and stability compared to persistent scatterer (PS) points. Addressing the challenge of sparse PS points involves filtering homogeneous points, optimizing the phase of distributed targets, mitigating noise influence on DS points, and subsequently deriving the deformation phase.

Methods for extracting homogeneous points encompass parametric hypothesis testing (e.g., GLRT algorithm), nonparametric hypothesis testing (e.g., BWS method), and efficient algorithms such as FaSHPS. However, due to the susceptibility of homogeneous points to spatial and temporal incoherence, it’s essential to optimize their phase. Common approaches for phase optimization include the maximum likelihood estimation method and coherence matrix decomposition.

In this study, the FaSHPS method is used for homogeneous point identification and the coherence matrix decomposition method is used to process the phase information. Once the phase information of DS points is obtained, phase untangling is carried out, followed by solving for the deformation phase, integrating both DS and PS points for a comprehensive deformation analysis [20].

### 2.4. Overview of the Study Area

The Jiangsu coastal highway holds significant importance as part of the national highway network, specifically the Shenyang to Haikou highway. It plays a pivotal role in Jiangsu Province’s transportation infrastructure, serving as a crucial component of the “fifteen shot six vertical and ten horizontal” highway framework, particularly in the “longitudinal” segment. Spanning a length of 318.376 km, the highway features an array of bridges, totaling 334 in number.

Designed to six-lane highway standards throughout its entirety, the G15 Coastal Highway is divided into two main sections: Lianyan and Yantong. The construction of the Yancheng to Nantong section was completed and opened to traffic in November 2005, while the Lianyungang to Yancheng section followed suit in October 2006. Notably, the G15 Coastal Highway closely skirts the Yellow River.

The highway’s proximity to the Yellow Sea situates most of its route within a marine sedimentary plain. The geological conditions in the region are intricate, characterized by low-lying terrain, an intricate river network, numerous lakes and ponds, and a high water table. Soft soil predominates in the area, often comprising thick or super-thick layers intersecting land and sea. This soil type exhibits high water content, natural porosity, compressibility, and low cohesion and consolidation coefficients—common traits along the coastal highway.

The soft soil layer serves as a shallowly buried deep soft soil foundation, presenting poor engineering properties. Consequently, construction necessitates foundation treatment methods, primarily employing pre-compression consolidation and powder spraying pile reinforcement. Despite these efforts, roadbed deformation issues have recurred during construction, posing challenges.

Since its inception, the Coastal Expressway has experienced settlement and uplift deformation across various sections, impacting driving safety and comfort. Addressing these challenges requires ongoing monitoring and maintenance efforts to ensure the highway’s long-term reliability and safety for motorists.

### 2.5. Data Presentation

**Figure 1:**
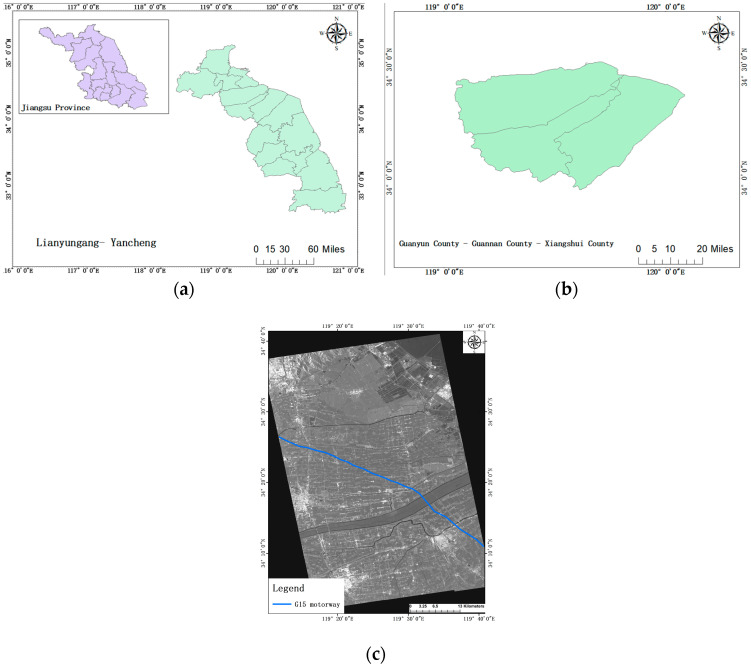
Geographic location of the study area: (**a**) Jiangsu Province and Lianyungang city–Yancheng city (**b**) Guanyun Country–Guannan Country–Xiangshui country (**c**) G15 motorway.

The focus of this project lies in observing the settlement dynamics within the K843+699-K887+991.5 section, spanning from Guanyun North Junction to Guanhe Bridge. To account for variations in surface linear structures, the observation area extends three hundred meters on both sides of the highway. This approach aims to derive the four-year average annual rate of subsidence from 2018 to 2021.The geographic location of the study area is shown in Figure 1.

The study utilizes image data from the Sentinel-1A satellite, which operates in the C-band and features synthetic aperture radar (SAR) capabilities. Specifically, the IW (Interferometric Wide swath) mode is employed to capture 250-km-long data with a spatial resolution of 5 m by 20 m (monovision). The imagery spans the timeframe of 2018 to 2021, encompassing a total of 73 SAR images.

Various methodologies and parameters necessitate meticulous selection and configuration during the construction of PS InSAR, DS InSAR, and SBAS InSAR models to capture deformation information accurately.

In the construction of the PS-InSAR technique model, the image dated December 31, 2019, was designated as the primary image from a pool of 73 images, while the remaining images served as secondary, resulting in 72 interferometric pairs. Subsequently, PS points were identified using the amplitude deviation amplitude divergence method. Phase unwrapping was executed via the least-cost flow algorithm, flat terrain correction was achieved utilizing SRTM data, and atmospheric delay errors were rectified employing quadratic polynomials.

Regarding the modeling of SBAS InSAR and DS InSAR techniques, a temporal baseline of 36 days was adopted for the interferometric pairs, with a de-entanglement threshold set at 0.4. A filtering window size of 15*15 was applied, and additional correlation among neighboring points was performed through DS-Insar.

The comprehensive commercial software developed by GAMMA Remote Sensing, Switzerland, specializing in interferometric radar data processing, was selected for this study.

**Figure 2:**
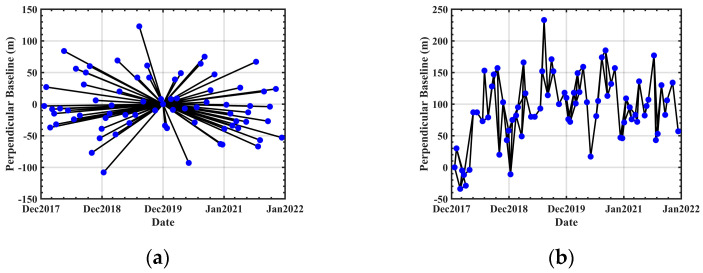
Spatiotemporal baseline plots: (**a**) PS-InSAR (**b**) SBAS-InSAR and DS-InSAR.

The spatiotemporal baseline plots illustrating the three techniques are depicted in Figure 2.

## 3. Results

The settlement results obtained from InSAR technology in this study are in the satellite line-of-sight (LOS) direction, which can be converted to the vertical direction based on the radar’s incidence angle. Considering the geometric relationship of radar side-view imaging and the correlation between LOS deformation observed by InSAR and surface three-component deformation, the vertical deformation data acquired from interferometry technology contributes more than 90% to the observation of satellite line-of-sight data [21,22].

### 3.1. Deformation Results Based on PS-InSAR Technique

**Figure 3:**
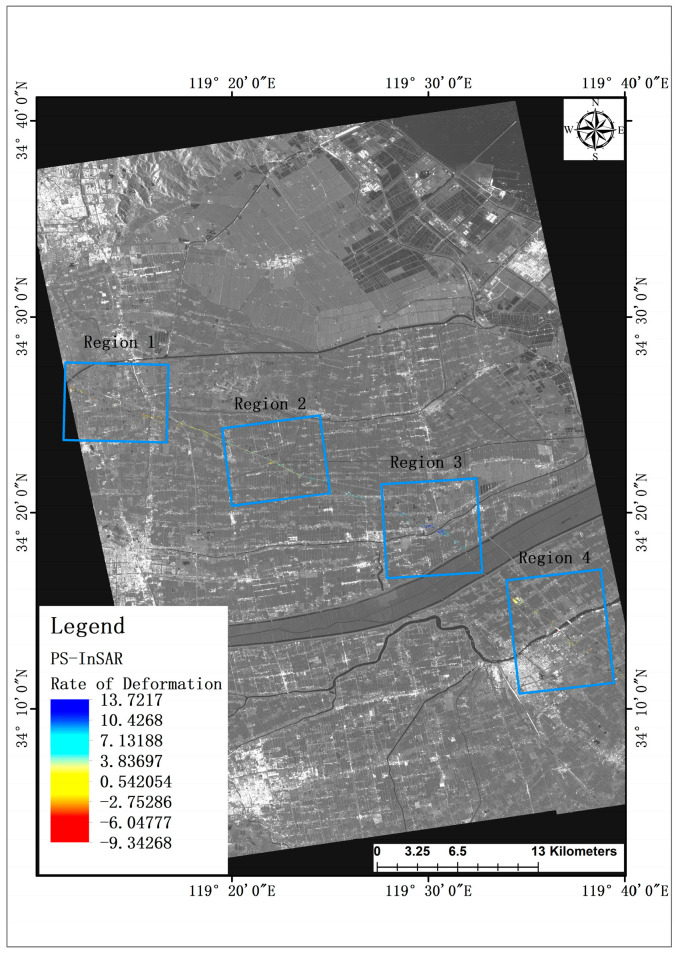
PS-InSAR based deformation rate map of the study area.

In the utilization of the PS-InSAR technology for image processing, a time-centered SAR image captured during sunny winter conditions was designated as the primary dataset. The amplitude deviation thresholding method was subsequently employed to effectively screen and isolate Persistent Scatterer (PS) points. A total of 2085 PS points were identified within the observation section along the G15 highway. Following the extraction of deformation phases from the PS points, deformation rate maps were generated for the two study areas. To facilitate a comprehensive analysis, the observation sections were partitioned into four regions each, based on the magnitude of deformation and the density of PS points. The ensuing analysis delves into the spatial distribution of deformation within each region, as depicted in Figure 3 below.

**Figure 4:**
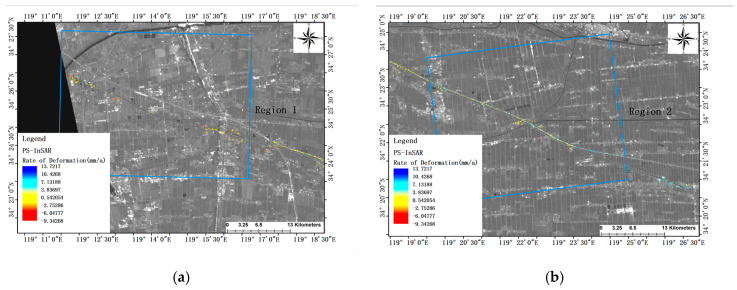

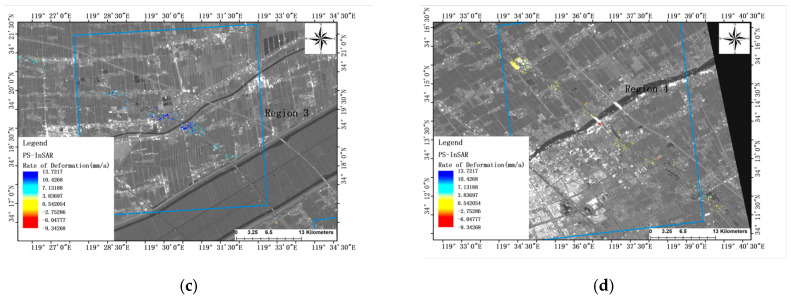
Deformation rate map of PS-InSAR observation focus area of G15 highway: (**a**) Region 1 (**b**) Region 2 (**c**) Region 3 (**d**) Region 4.

Due to the limited number of Persistent Scatterer (PS) points acquired through the PS-InSAR technique, the visualization on the global map is compromised. Consequently, a closer examination of G15 highway observation regions 1–4 is warranted, as depicted in Figure 4. Upon extracting pertinent deformation data, it becomes evident that settlement predominates within regions 1 and 4 of the G15 observation section. Notably, region 4 exhibits a maximum subsidence rate of 9.3 mm/a, while the respective average subsidence rates for the two regions stand at 2.3 mm/a and 2.5 mm/a. Conversely, region 3 experiences predominantly uplift, with no instances of settlement detected among the PS points across the entire area. The maximum lifting rate within this region reaches 13.7 mm/a, with an average lifting rate of 9.8 mm/a. Furthermore, area 2 exhibits more pronounced settlement and uplift phenomena, with a maximum settlement rate of 7 mm/a and a maximum lifting rate of 8.7 mm/a.

### 3.2. Deformation Results Based on SBAS-InSAR Technique

**Figure 5:**
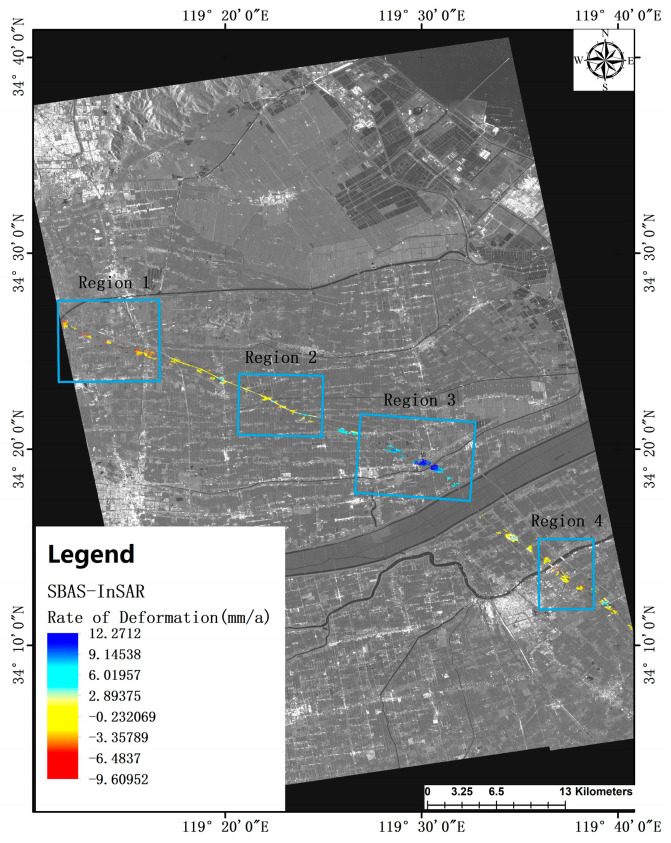
SBAS-InSAR based deformation rate map of the study area.

The SBAS-InSAR technique has proven to be more effective in acquiring coherent points compared to the PS points. Specifically, it was determined that SBAS acquired a total of 13,781 observation points within the freeway observation segment under study. The analysis of these observation points was conducted by partitioning the two observation sections according to specific principles, and the resulting deformation rate maps are depicted in Figure 5.

Upon examining the deformation of coherent points within each region of the G15 highway observation section, certain patterns emerged. Regions 1 and 4 were predominantly characterized by subsidence, with maximum subsidence rates of 9.6 mm/a and 6.8 mm/a, respectively. These subsidence rates significantly outweighed the maximum uplift rates observed in these regions. The average subsidence rates for the subsided points were calculated to be 3.4 mm/a and 2 mm/a for regions 1 and 4, respectively. In contrast, the average uplift rates for points within these regions were all less than 1 mm/a.

In area 2, notable deformation of both subsidence and uplift concurrently occurred, with maximum rates of 7.9 mm/a and 5.3 mm/a, respectively. Interestingly, the average rates of subsidence and uplift were found to be similar, each approximately 2 mm/a.

The internal dynamics of area 3 presented a unique scenario, as no coherent points indicating subsidence and deformation were identified. Instead, the average internal uplift rate for this area was calculated to be 8.8 mm/a, with the maximum uplift rate of points reaching 12.3 mm/a.

### 3.3. Deformation Results Based on DS-InSAR Technique

**Figure 6:**
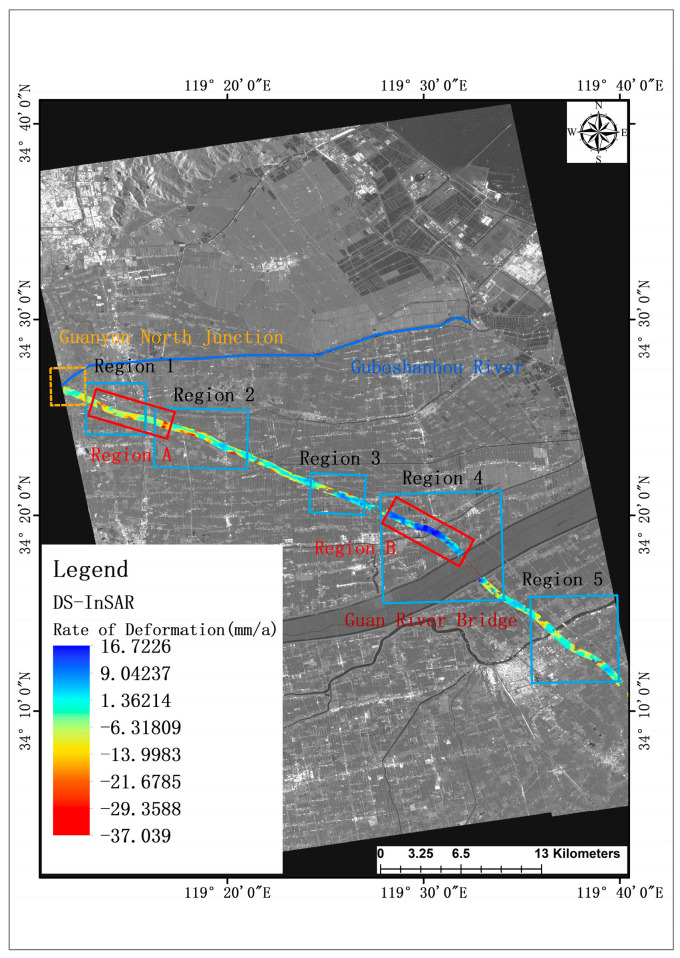
DS-InSAR-based deformation rate map of the study area.

The limitations of PS-InSAR technology, stemming from its inherent characteristics, result in a small number of persistent scatterer points being identified on road observation sections. This constraint makes it challenging to accurately capture the intricate and continuous deformation information along the road segment. In contrast, the DS-InSAR technique, which targets distributed scattering points, offers the advantage of acquiring a larger quantity of points during road deformation observation. In the current study, the DS-InSAR technique, aided by the FaSHPS fast homogeneous point algorithm, successfully obtained 63,394 distributed scattering points on the road observation segments. Following a similar methodology to the previous section, the observation segments were partitioned based on zoning principles, and the resulting deformation rate map, incorporating zoning information, is depicted in Figure 6. This approach allows for a more comprehensive analysis, focusing on key deformation areas and providing insights into the dynamics of the road segment under observation.

Analyzing the five zones in the deformation rate map obtained based on the DS-InSAR technique, it can be found that the DS-InSAR observation results are somewhat similar to the SBAS-InSAR results, but supplemented with richer deformation information. Specifically, there are more serious subsidence conditions in all five sub-regions, with the maximum annual average subsidence exceeding 20 mm. The average subsidence rates inside Regions 1, 2, and 3 are 8.8 mm/a, 9.2 mm/a, and 7.2 mm/a, respectively. The problem of uplift inside Regions 3 and 4 is also more serious, with the average uplift rates of 4.9 mm/a, 8.8 mm/a, and the maximum uplift rates exceeding 10 mm/a, among which Regions 3 and 4 are more serious, with the average uplift rates of 4.9 mm/a, 8.8 mm/a, and the maximum uplift rates exceeding 10 mm/a. The maximum uplift rate is more than 10 mm/a, of which area 4 reaches 16.7 mm/a.

In summary, the results of highway deformation monitoring based on DS-InSAR are similar to those of SBAS-InSAR in general trend, but there are some differences in the specific values, and there are large inhomogeneous deformations in the results of DS-InSAR technology, which have great influence on the performance and safety of the road, and may represent serious diseases within the structure. There are more serious diseases, that need to be monitored and fieldworked.

## 4. Discussion

### 4.1. Analysis of Results

In the preceding section, the primary deformations within the G15 highway study area were delineated by segmenting the deformation rate maps generated through three distinct techniques. While the observation points acquired by these techniques differ, resulting in variations in partitioning, they all identify several focal areas in common. Notably, all three InSAR techniques employed in the deformation monitoring of the G15 highway study segment detected a distinct subsidence area (referred to as Area A) and a consistent uplift area (referred to as Area B), as depicted in the deformation rate map generated by DS-InSAR, illustrated in Figure 6.

According to the “Tianlou Guan River Special Bridge Engineering Geological Investigation Report” [23] issued by the China Railway Fifth Survey and Design Institute in 2009, the study section of the river lies within the Yellow-Huaikai alluvial plain. Characterized by a flat topography, the region features a dense river network and a slight southwest to northeast inclination towards the Yellow Sea. The stratigraphy of the area comprises artificial fill, Holocene alluvial clay, chalk, marine soft clay of the Quaternary system, and Upper Pleistocene alluvial and flooding clay and chalky clay of the Quaternary system. The peak acceleration of ground vibration within the region is measured at 0.05 g, indicating a seismic intensity of 6 degrees. The ground vibration response spectrum exhibits characteristic cycle zoning of three zones. The area is characterized by abundant groundwater with high water levels, with the depth of the groundwater level ranging from 0.0 m to 2.5 m during the survey period. Consequently, it can be inferred that the area lacks adverse geological structures.

The specific analyses conducted on Areas A and B reveal that the deformation proximate to Area A primarily arises from combined and concentrated loads, notably including the Guanyun North Junction, the G15 motorway, and the Lianyan Railway. Additionally, this region is impacted by agricultural Guan practices, along with erosion and sedimentation processes associated with the Gupo Shanhou River. Particularly, discernible erosion effects are observed along the river’s south bank. The collective influence of these factors culminates in settlement deformation within the vicinity of Area A.

Conversely, the construction and operational activities involving roads, railways, and railway bridges in the vicinity of Area B are subject to the influence, erosion, and sedimentation dynamics of the adjoining river. Notably, the left bank of the river, corresponding to the north side, primarily contends with sedimentation phenomena. Moreover, the elevated groundwater level necessitates careful consideration regarding the structural foundation’s susceptibility to both erosion and the supportive effects of groundwater.

Based on the findings outlined in the “Tianlou Guan River Bridge Engineering Geological Survey Report” and “Code for Geology Investigation of Railway Engineering (TB10012-2019)” [24] concerning the assessment of environmental water erosion (inclusive of surface water and groundwater) on construction materials, several pertinent observations emerge. Firstly, there exists groundwater sulfate erosion of construction materials within this vicinity, classified at environmental role level H1 (200 ≤ SO42- ≤ 1000). Additionally, chloride salt erosion affects steel reinforcement, also designated at environmental role level H1. Furthermore, chloride salt erosion extends to both steel reinforcement and the foundation of structures, with chloride erosion of steel reinforcement rated at environmental effect level L2, and chloride erosion of steel reinforcement by surface water rated at environmental effect level L1. These assessments collectively suggest that while environmental water erosion does occur, its impact appears to be relatively moderate.

Furthermore, despite the influence of traffic loads and other contributing factors in both areas under study, the magnitude of these loads within this specific area, particularly when contrasted with the more concentrated loads present in Area A, is comparatively lesser. Considering these various factors holistically, it is reasonable to anticipate a greater degree of uplift deformation in the vicinity of this area, notably accentuated on the north side of Area B.

In this study, data within 200 m on each side of the road median were collected and analyzed to study the deformation of the main observation area. A road is a linear strip-shaped three-dimensional structure, primarily integrated with the ground, where ground deformation significantly impacts the highway. Therefore, to further analyze road deformation in the study area, it’s crucial to integrate the results of ground deformation from a larger surrounding area.

**Figure 7:**
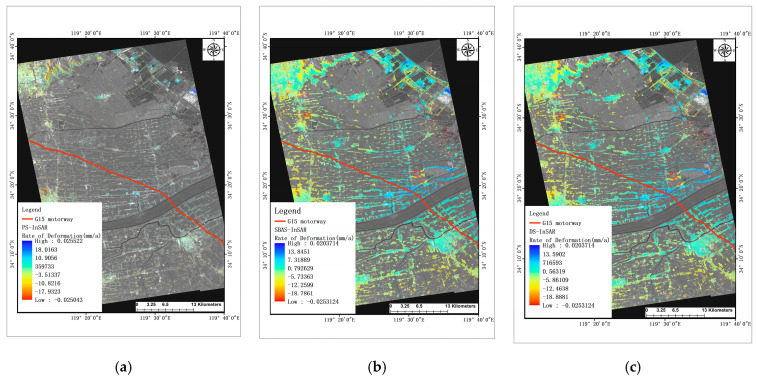
Ground deformation information in the vicinity of the study area: (**a**) PS-InSAR (**b**) SBAS-InSAR (**c**) DS-InSAR.

In Figure 7 below, the ground area near the observation zone selected for this study is depicted. Similar to the previous section, deformation observations were conducted in this area using the three InSAR techniques.

It is evident that the predominant deformation in the ground area near the highway, as selected for this study, is subsidence. Specifically, in the observation area extending from northwest to southeast of Lianyungang City’s Haizhou District, encompassing Xiaoyi Township, Getting Off the Bus, Yangjizhen Township, and Xiangshui County near the studied road section. Initially, deformation along the road direction is mainly characterized by subsidence, transitioning to uplift deformation towards the vicinity of the irrigation river, and then returning to subsidence deformation as we move towards Xiangshui County in the southeast. Deformation trends are more pronounced near towns, roads, and bridges, with less data collected in the cultivated areas adjacent to the highway, resulting in a lower deformation rate.

In Figure 7, after filtering out a few points with excessive deformation located far from the road, the observation results from the three InSAR techniques collectively indicate that Haizhou District in Lianyungang City, specifically the upper-left part of the figure, experiences significant settlement and deformation. The maximum cumulative settlement exceeds 110 mm, with a peak settlement and deformation rate of 31.2 mm per year during the observation period. Additionally, extensive areas exhibit settlement rates of 20 mm per year or higher in this region. Near area A in Figure 6, both Xiaoyi Town and Xiaoche Town are characterized by settlement deformation dominance. Notably, this area hosts key transportation hubs like Lianyungang Huaguoshan International Airport and Guanyun North Hub, leading to higher traffic loads. The maximum settlement rate near the airport reaches 28.07 mm per year, while near Guanyun North Hub, it exceeds 10 mm per year. Given the proximity to area A, road deformation within this region is more pronounced, significantly impacting road infrastructure, aligning with our previous analysis.

In the vicinity of area B in Figure 6, specifically near the north and south banks of the irrigation river, there are noticeable areas of uplift deformation distributed along the riverbanks. Near the north bank of the irrigation river, the maximum cumulative uplift deformation during the observation period exceeds 45 mm, with a maximum uplift deformation rate reaching 12.1 mm per year. Ground deformation rates near Rongshui County are relatively smaller, characterized mainly by minor uplift deformations in the county center, with deformation rates mostly below 5 mm per year. However, in the suburbs, there are identifiable areas of subsidence deformation, with the maximum subsidence rate being approximately 10.5 mm per year.

Overall, the three InSAR techniques yield similar observations regarding ground deformation along the G15 highway. Among them, the SBAS-InSAR and DS-InSAR results exhibit greater numerical consistency and provide more extensive data, aligning well with the observed road deformation data. In contrast, the analysis of PS-InSAR results alongside road deformation observations proves challenging due to the limited number of observation points obtained. Consequently, interpreting PS-InSAR results alongside road deformation data is hindered by the scarcity of observation points.

### 4.2. Validation of Results

**Figure 8:**
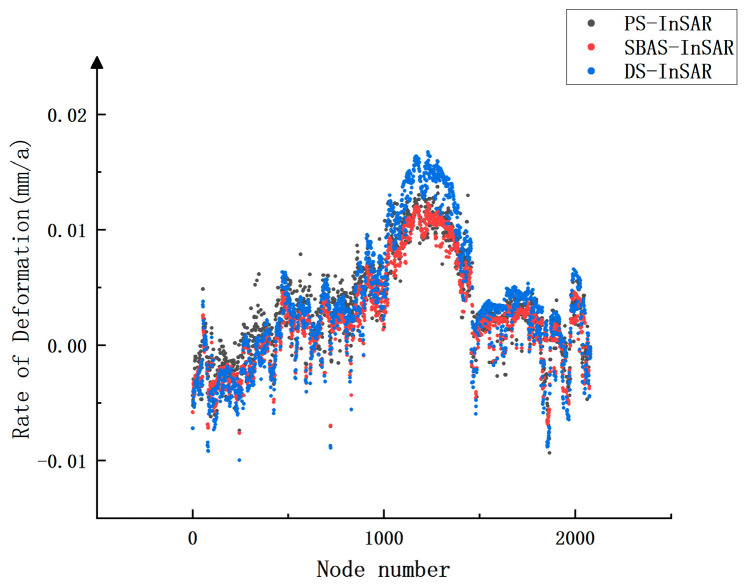
Deformation rate of the common observation point of the three InSAR techniques.

To ascertain the reliability of road deformation monitoring through the implementation of various Interferometric Synthetic Aperture Radar (InSAR) techniques, this study conducts an examination utilizing data from 2082 observation points. These observation points are integral components of the results derived from all three InSAR techniques employed. The findings of this analysis are presented in Figure 8.

Table 1 presents the outcomes of correlation analyses conducted on the three InSAR techniques, employing the Pearson correlation coefficient to signify the interrelationship among the respective results. The correlation coefficient between SBAS-InSAR and PS-InSAR is measured at 0.948, while the correlation coefficient with DS-InSAR stands at 1, demonstrating statistical significance at the 0.01 level. Upon amalgamating the findings from Figure 8 and Table 1, it becomes apparent that the correlation between SBAS-InSAR and DS-InSAR observations exhibits exceptional strength, along with a robust correlation with the observations derived from the PS-InSAR technique.

To delve deeper into the numerical variance of the observed values obtained through the three techniques, Euclidean distances between the respective data sets within the three groups were computed, as outlined in Table 2. The findings indicate that the average discrepancy between the observed values of any two techniques is under 2 mm/a. Particularly in regions exhibiting substantial deformation, the impact of this disparity is less pronounced. Consequently, it is reasonable to assert that the observed results derived from the three techniques are fundamentally analogous and can be mutually referenced.

## 5. Conclusions

In this study, the PS-InSAR, DS-InSAR, and SBAS-InSAR techniques were employed to monitor the deformation of the designated segment of the G15 motorway over a span of four years. Subsequently, the findings were meticulously summarized and analyzed, yielding the following primary outcomes and conclusions:

1. The results obtained from each technique across distinct observation areas are delineated. Within the surveyed stretch of the G15 motorway, certain regions exhibit an average deformation rate exceeding 5 mm/a, with the maximum deformation rate surpassing 10 mm/a. Such findings underscore the imperative need for vigilant monitoring and potential remediation efforts to mitigate potential issues.

2. Concomitantly considering the actual geo-geological and hydrological factors, an analysis of the causative factors behind deformation within the four focal areas was conducted. Factors such as river hydrology and geological structures predominantly influence the observation area; however, a more nuanced examination of underlying causes necessitates supplementary field research data.

3. The observational outcomes yielded by the three InSAR techniques were juxtaposed through correlation and difference analyses. Notably, the results evince minimal disparities among the techniques’ observations, thereby affirming their accuracy in the domain of road deformation detection.

This study presents crucial data and analysis findings for comprehending the deformation characteristics and underlying causes along the observed section of the G15 highway. In subsequent research applications, risk zone identification will rely on the conclusions drawn from InSAR technology data. Subsequently, ground-penetrating radar, leveling, and other methods will be employed to procure more precise and detailed deformation information, aiding in the identification of causative factors. This approach aims to support decision-making in road maintenance and contribute to the effective operation and management of the highway. Additionally, the comparative assessment of deformation monitoring technologies holds significant reference value, offering guidance and insights for further research and practical applications in related fields.

## Figures and Tables

**Table 1:** Pearson correlation coefficients for the three InSAR techniques.

Technique	SBAS-InSAR	PS-InSAR	DS-InSAR
SBAS-InSAR	1		
PS-InSAR	0.948	1	
DS-InSAR	1	0.948	1

**Table 2:** Euclidean distance averages for the three InSAR techniques.

	SBAS-InSAR	PS-InSAR	DS-InSAR
Average (mm/a)	1.22	1.41	1.73

## Data Availability

Data are contained within the article.
